# The Avon Breast Screening Programme

**Published:** 1989-05

**Authors:** Zoë Davies

**Affiliations:** Consultant Radiologist, Bristol Royal Infirmary


### The Avon Breast Screening Programme

**Zoë Davies**
Consultant Radiologist, Bristol Royal Infirmary

The Forrest Report on Breast Cancer Screening was published in the Spring of 1987 and, as a result of its recommendations, a countrywide National Health Service Breast Screening Programme is being instigated. The Committee was chaired by Sir Patrick Forrest and included other Surgeons, Radiologists, a Radiotherapist, a Specialist in Community Medicine, a Histopathologist and a member from the Health Economics Department and various observers and participating experts.

### SCREENING TRIALS

The Committee studied a number of overseas trials of breast screening, particularly the Health Insurance Programme from New York and the more recent Two Counties Trial in Sweden and trials in Holland. Reports from these trials record up to 30% reduction in the mortality from breast cancer for ten years, concentrated in women aged fifty and over. The effect on mortality in the forty- forty-nine age group is still uncertain. The evidence from these trials led the committee to conclude that breast screening can reduce the mortality from breast cancer and this was, therefore, recommended.

The whole matter then took on a political significance and it was decided to implement a countrywide screening programme within a very short period, (i.e. by 1990) considering the limited number of staff and facilities available so to do. There is no countrywide breast screening programme to provide guidelines for the U.K., this being much more complex than organising a trial such as has been taking place in the U.K. since 1979.

### TRAINING IN THE UNITED KINGDOM

The Jarvis Screening Centre in Guildford and the Edinburgh Centre were the main centres for breast screening by mammography in the U.K. trial. In England and Wales the Jarvis Centre has become the first training centre for the national undertaking. The Jarvis Centre has trained centres in Manchester, Nottingham and King's College, London, and these, in turn, will, with Guildford, be responsible for the training required throughout the regions in England. Wales and Scotland will undertake their own training. Radiographers and Radiologists have to be trained in this specialised field, as do the Histo- and Cytopathologists. The Surgeons and Radiotherapists have to decide on the appropriate form of treatment for the often small lesions found during screening. It is recognised that, for the whole endeavour to succeed, extremely high standards have to be achieved and monitored through carefully planned quality assurance programmes, from identifying the patients, through to treatment of any discovered lesions. It was intended that the four training centres, and one centre per region should be running by Spring, 1988, and that the remaining centres should be developed over the following two years. Thus, the first centre in the South West is in Cornwall, based at the Treliske Hospital.

Detailed training programmes for Radiographers and Radiologists have been produced by the College of Radiographers and the Royal College of Radiologists and are under way. The Royal College of Pathologists and the British Association of Surgical Oncology are producing guidelines for their involved members. Computer programmes are being developed for recording results and training plans for office and clerical staff are being organised. Medical Physicists have a considerable role to play in the scheme, particularly in maintaining the quality control requirements of the equipment.

### THE SCREENING PROCEDURE

The screening procedure is divided into two phases, the first being the basic screen, and the second the assessment stage, when abnormalities seen on the basic screen mammograms are sorted out. Those women found to have no significant abnormalities on assessment are returned to the normal call/recall system; those with genuine abnormalities proceeding to confirmed diagnosis and treatment. No perfect screening test for breast cancer has yet been discovered, but the best so far has been shown to be mammography— x-rays of the breast taken with special techniques suitable for examining soft tissues. This requires dedicated x-ray machines and film processors and specially developed films, cassettes and grids. It has been decided that the basic screen should consist of a single medio-lateral oblique view of each breast, with no physical examination at this stage. At the assessment stage further x-ray films will be taken, including cranio-caudal, true lateral and localised views, with magnification views if indicated. The women will have a physical examination of the breast, ultrasound examination of local lesions and fine needle aspiration of palpable lesions. If a problem of diagnosis remains after these procedures, the women will be referred to a small number of Surgeons with a particular interest in the field, for biopsy with localisation procedures as necessary. The discovered cancers will then be treated by surgery and radiotherapy, according to their size, histological type and position.

All women in the U.K. between the ages of fifty and sixty-four are eligible to be screened and, in Avon, there will be approximately 67,000 women in this age band. They are to be offered single view mammography every three years. After sixty-four they may continue to be screened regularly if they wish it, but they will not be called up for it routinely, as response has been shown to be poor in the older age groups.

It is likely that approximately 70% of invited women will respond to the invitation for mammography, approximately 20,000 women being screened per year. Approximately 10% of these women will need to have further assessment. The majority of women in the 10% will be found to fall within the normal group and a small proportion will need surgical biopsy to separate benign from malignant lesions. From the basic annual screen of about 20,000 women approximately 100 cancers are likely to be picked up per year of the first screening cycle, i.e. in the region of five per thousand.

### AVON BREAST SCREENING UNIT

The three Health Districts in Avon make up just under two Forrest basic screening units. It has been decided by the three Health Districts that the screening programme should be run as a single entity, organised through the Bristol and Weston District. The Forrest Committee recommended that, where possible, the basic screening unit should not be within a hospital dealing with symptomatic women, as the majority of women to be screened would be perfectly fit and would appreciate less clinical surroundings.

It is hoped that the Avon Breast Screening Unit will be running by late Summer, 1989, based in the Central Health Clinic in refurbished space previously occupied by the Chest Clinic. The basic screening facilities will comprise of a static x-ray unit in the Central Health Clinic on which women living in central Bristol will be examined, and a second x-ray unit housed in a mobile van which will visit the more peripheral areas of Avon, visiting approximately twelve sites over the three year period of the screening cycle. The assessment unit will also be situated in the Central Health Clinic, with more sophisticated radiological equipment and ultrasound equipment.

**THE ROLE OF THE F.P.C.**

The Family Practitioner Committee will produce the cohort of women from fifty to sixty-four and keep this up to date. It is planned to screen the women according to general practices. The screening unit office will send the General Practitioners lists of eligible patients. The G.P.'s will be asked to check that the addresses are correct and to remove temporarily or permanently the names of women for whom screening would be inappropriate, i.e. bilateral mastectomy or severe illness. The ammended lists will be returned to the screening office who will send out invitation appointments to the eligible women, with accompanying letters expressing their G.P.'s approval (if given), and with explanatory leaflets, including instructions for changing appointments and for finding the screening centres. The women and their G.P.'s will be informed of the result of the basic screen. Normal women will be informed of each stage. It is hoped that, with G.P.'s approval, the majority of impalpable lesions demonstrated on mammography will be referred to a limited number of Surgeons with a special interest in this work.

**IMPLEMENTING THE PROGRAMME**

The programme cannot succeed unless a high proportion, at least 70%, of women who are invited actually accept the invitation to be screened. This requires education and publicity at the appropriate time for the women, their families and friends, and to the primary care teams. In Avon we have a Health Education Officer to help us promote the project through the initial phases. The breast screening staff will rely enormously on the help and co-operation of the General Practitioners and their staff and it is intended to visit each individual practice or group of practices approximately three months before the women are to be called for screening, to explain the procedure and to distribute information packs and a video which has been developed for the primary care teams.

It has to be recognised that we shall not be able to pick up every cancer at an early stage and that some women will present with symptomatic breast cancer between screening visits. These facts have to be presented honestly to women whilst still encouraging them to attend for screening.

